# Hospitalized adult patients with 2009 influenza A(H1N1) in Beijing, China: risk factors for hospital mortality

**DOI:** 10.1186/1471-2334-10-256

**Published:** 2010-08-27

**Authors:** Xiuming Xi, Yuan Xu, Li Jiang, Ang Li, Jie Duan, Bin Du

**Affiliations:** 1 Department of Critical Care Medicine, Beijing Fuxing Hospital, Capital University of Medical Sciences, 20A Fu Xing Men Wai Da Jie, Beijing 100038, China; 2 Department of Critical Care Medicine, Beijing Tongren Hospital, Capital University of Medical Sciences, 1 Dong Jiao Min Xiang, Beijing 100730, China; 3 Department of Critical Care Medicine, Beijing Friendship Hospital, Capital University of Medical Sciences, 95 Yong An Lu, Beijing 100050, China; 4 Beijing Bureau of Health, Block B, 70 Zao Lin Qian Jie, Beijing 100053, China; 5 Medical ICU, Peking Union Medical College Hospital, Peking Union Medical College, 1 Shuai Fu Yuan, Beijing 100730, China

## Abstract

**Background:**

In April 2009, the pandemic influenza A(H1N1) virus emerged and spread globally. The objective of this study was to describe the independent risk factors for hospital mortality and the treatment effect of corticosteroids among patients with 2009 influenza A(H1N1) infection.

**Methods:**

We retrospectively obtained clinical data of 155 adult patients with confirmed infection of 2009 influenza A(H1N1) in 23 hospitals in Beijing, China from October 1 to December 23, 2009. Risk factors for hospital mortality were identified with multivariate logistic regression analysis.

**Results:**

Among the 155 patients, 90 (58.1%) were male, and mean age was 43.0 ± 18.6 years, and comorbidities were present in 81 (52.3%) patients. The most common organ dysfunctions included acute respiratory failure, altered mental status, septic shock, and acute renal failure. Oseltamivir was initiated in 125 patients (80.6%), only 16 patients received antiviral therapy within 48 hours after symptom onset. Fifty-two patients (33.5%) were treated with systemic corticosteroids, with a median daily dose of 80 mg. Twenty-seven patients (17.4%) died during hospital stay. Diabetes [odds ratio (OR) 8.830, 95% confidence interval [CI] 2.041 to 38.201, p = 0.004) and lactate dehydrogenase (LDH) level (OR 1.240, 95% CI 1.025 to 1.500, p = 0.027) were independent risk factors of hospital death, as were septic shock and altered mental status. Corticosteroids use was associated with a trend toward higher hospital mortality (OR 3.668, 95% CI 0.987 to 13.640, p = 0.052).

**Conclusions:**

Hospitalized patients with 2009 H1N1 influenza had relative poor outcome. The risk factors at hospitalization may help clinicians to identify the high-risk patients. In addition, corticosteroids use should not be regarded as routine pharmacologic therapy.

## Background

Since the emergence of the novel 2009 influenza A(H1N1) in March 2009 [1], the world has witnessed its rapid and global spread to almost all countries and territories from April to June 2009 [2,3], which resulted in the declaration of the first phase 6 global influenza pandemic by the World Health Organization (WHO) on June 11, 2009 [4].

A variable proportion of patients with 2009 influenza A(H1N1) infection were hospitalized [5]. Although clinical manifestations and outcome of these patients have been described, the reported cohorts often include both inpatients and outpatients [6], or both adult and pediatric patients [7,8], or all cases with confirmed, suspected, and probable diagnoses [8-10]. The heterogeneous patient population in the above studies might preclude the possibility of reporting valid risk factors for mortality.

We report on 155 adult hospitalized patients with confirmed infection of 2009 influenza A(H1N1) in Beijing, China from October 1 to December 23, 2009. The objective of this retrospective study was to describe the independent risk factors for hospital mortality among these patients. Moreover, the factors influencing corticosteroids treatment as well as its effect on hospital mortality were also evaluated.

## Methods

The first case of 2009 influenza A(H1N1) in mainland China was identified on May 11, 2009 [8]. As a result, all hospitals were required to report every case to local health authorities and Center for Disease Control (CDC). In this retrospective study, cases were captured through the records in Beijing Health Bureau and Beijing CDC. All hospitals with H1N1 patients were identified and contacted for possible participation into the study. Twenty-three hospitals provided positive feedback, and constituted the study group.

Patients were eligible if they were (1) ≥ 18 years old; (2) admitted to any of the 23 participating hospitals from October 1 to December 23, 2009; (3) diagnosed as confirmed 2009 influenza A(H1N1) infection, according to case definitions developed by the World Health Organization [11]. Specifically, a confirmed case was defined by a positive result of a real-time reverse-transcriptase-polymerase-chain-reaction (RT-PCR) assay performed at a laboratory operated under the auspices of the Chinese CDC [8].

Case report form was initially developed by one investigator (BD), and then cycled among all investigators for feedback and pilot testing until consensus was reached. All reviewers were trained during a 1.5-hour course before the study, and then dispatched to all participating hospitals to review case records of all eligible cases.

For all patients, the following data were recorded: sex, age, major diagnosis, comorbidities, clinical presentation at symptom onset and on hospital admission, major laboratory results on hospital admission, complications, antiviral and antibiotic treatment, supportive treatment, and outcome. In particular, septic shock was defined according to the consensus definition of American College of Chest Physicians/Society of Critical Care Medicine [12], while acute respiratory failure or acute renal failure was defined if sequential organ failure assessment score for that particular organ was greater than two points [13].

Data were entered into a Microsoft Excel database (Microsoft, Seattle, Wash., USA) by a data manager (BD) under the supervision of the study steering committee (XM, YX, LJ, AL). Data were checked for inconsistencies and logical errors on entry, and queries were sent to the source hospital for resolution.

The study protocol was approved by Institutional Review Board of Beijing Fuxing Hospital (IRB-2009-0135), and the need for informed consent was waived because of the retrospective study design.

Values are presented as the mean ± standard deviation or median (interquartile range) when appropriate (continuous variables), or as a percentage of the group from which they were derived (categorical variables). Continuous variables were compared with the use of the Student's t-test or Mann-Whitney test. The chi-square test or Fisher's exact test was used to compare categorical variables. For determination of independent predictors for hospital mortality, odds ratio was estimated on the basis of multivariate logistic regression analysis, including exploration for robustness and interactions using likelihood ratio tests. Two separate models using stepwise conditional forward entry were constructed - one for demography, comorbidities, clinical presentations, and laboratory tests on admission, and one for complications including organ failures and infections, if p < 0.1 in univariate analysis. Corticosteroids treatment was forced into the first model in order to examine the effect on patient outcome. Multivariate logistic regression analysis was also used to determine the independent predictors of corticosteroids treatment.

The Kaplan-Meier method was used to determine the probability of survival over the duration of follow-up and to generate survival curves, censoring at 30 days after symptom onset.

All comparisons were unpaired and all tests of significance were two-tailed. A p value < 0.05 was considered as statistically significant.

## Results

### Characteristics of study population

From October 1 to December 23, 2009, 562 patients with 2009 influenza A(H1N1) infection who required hospitalization were reported to Beijing Health Bureau, among whom 64 patients died. Of all these patients, 287 patients were admitted into any one of the 23 participating hospitals, accounting for 51.1% of all reported cases. After excluding 132 paediatric patients, 155 adult patients were included in data analysis.

Among the 155 adult patients with confirmed 2009 influenza A(H1N1) infection, 90 (58.1%) were male, and mean age was 43.0 ± 18.6 years. Eighty-one patients (52.3%) had at least one comorbidity, including cardiovascular disease, chronic lung disease, diabetes, cerebral vascular disease, and malignancy. In particular, 12 patients were pregnant women, and 2 patients were postpartum women (Table 1).

**Table 1:** Hospitalized adult patients with 2009 Influenza A (H1N1) in Beijing: demographics, comorbidities, and clinical presentation

	Survivor (n = 128)	Nonsurvivor (n = 27)	Total (n = 155)	P value
Male sex	73 (57.0%)	17 (63.0%)	90 (58.1%)	0.570
Age, year	42.4 ± 18.2	45.9 ± 20.7	43.0 ± 18.6	0.388
Ethnicity				
Han Chinese	127 (99.2%)	25 (92.6%)	152 (98.1%)	0.079
Smoker	28 (21.9%)	7 (25.9%)	35 (22.6%)	0.647
No comorbidity	60 (46.9%)	14 (51.9%)	74 (47.7%)	0.609
Comorbidity				
Cardiovascular disease	34 (26.6%)	11 (40.7%)	45 (29.0%)	0.140
Hypertension	25 (19.5%)	10 (37.0%)	35 (22.6%)	0.048
Ischemic heart disease	19 (14.9%)	5 (18.5%)	24 (15.5%)	0.572
Chronic lung disease	19 (14.9%)	3 (11.1%)	22 (14.2%)	0.768
COPD	8 (6.3%)	2 (7.4%)	10 (6.5%)	0.686
Diabetes	13 (10.2%)	7 (25.9%)	20 (12.9%)	0.051
Pregnancy or postpartum	12 (9.4%)	2 (7.4%)	14 (9.0%)	1.000
Pregnancy	11 (8.6%)	1 (3.7%)	12 (7.7%)	0.693
Postpartum < 1 month	1 (0.8%)	1 (3.7%)	2 (1.3%)	0.319
Cerebral vascular disease	6 (4.7%)	3 (11.1%)	9 (5.8%)	0.191
Symptoms at disease onset				
Fever (> 37.8°C)	121 (94.5%)	24 (88.9%)	145 (93.5%)	0.380
Cough	117 (91.4%)	25 (92.6%)	142 (91.6%)	1.000
Dyspnea	56 (43.8%)	17 (63.0%)	73 (47.1%)	0.069
Sore throat	42 (32.8%)	4 (14.8%)	46 (29.7%)	0.063
Myalgia	24 (18.8%)	3 (11.1%)	27 (17.4%)	0.416
Rhinorrhea	21 (16.4%)	3 (11.1%)	24 (15.5%)	0.770
Hemoptysis	15 (11.7%)	4 (14.8%)	19 (12.3%)	0.746
Diarrhea	6 (4.7%)	1 (3.7%)	7 (4.5%)	1.000
Vomiting	5 (3.9%)	0	5 (3.2%)	0.588
Time from symptoms to hospital admission, day*	5.0 (3.0, 7.0)	6.0 (3.5, 7.0)	5.0 (3.0, 7.0)	0.376
Clinical presentation on admission				
Dyspnea	61 (47.7%)	21 (77.8%)	82 (52.9%)	0.004
Pneumonia on CXR	104 (81.3%)	25 (92.6%)	129 (83.2%)	0.254
Laboratory tests on admission				
White cell count, 10^9^/L	6.1 ± 3.7	7.3 ± 5.1	6.3 ± 4.0	0.183
Lymphocyte, 10^9^/L	1.2 ± 1.4	0.7 ± 0.3	1.1 ± 1.3	0.118
CPK, U/L	339 ± 620	538 ± 548	373 ± 610	0.222
CK-MB, μg/L	23 ± 33	29 ± 22	24 ± 31	0.457
cTnI, μg/L	0.16 ± 0.23	0.32 ± 0.69	0.21 ± 0.43	0.417
LDH, U/L	356 ± 240	744 ± 759	432 ± 422	0.041
Total bilirubin, μmol/L	20.2 ± 57.8	48.8 ± 156.6	25.8 ± 86.2	0.431
AST, U/L	57 ± 49	348 ± 803	113 ± 366	0.105
ALT, U/L	51 ± 111	123 ± 254	65 ± 152	0.209

ALT, alanine transaminase; AST, aspartate transaminase; CK-MB, creatine kinase-MB; COPD, chronic obstructive pulmonary disease; CPK, creatine phosphokinase; cTnI, cardiac troponin I; CXR, chest-X ray; LDH, lactate dehydrogenase

*expressed as median (interquartile range)

The most common presenting symptom was fever (> 37.8°C) (93.5%), followed by cough (91.6%), dyspnea (47.1%), sore throat (29.7%), and myalgia (17.4%). The median time from symptom onset to hospital admission was 5 days. On hospital admission, 82 patients (52.9%) still complained dyspnea, and 129 patients (83.2%) were diagnosed as pneumonia according to chest X-ray. Forty of 141 patients (28.4%) who were tested had leukopenia (white cell count < 4.0 × 10^9^/L), while 19 patients (13.5%) had leukocytosis (white cell count > 10.0 × 10^9^/L), and 53 of 125 patients (42.4%) had lymphopenia (lymphocyte count < 0.8 × 10^9^/L). Laboratory tests also suggested mild to moderately increased cardiac enzymes, liver enzymes, and total bilirubin (Table 1). Of 126 patients who were tested, 74 patients (58.7%) had elevated level of lactate dehydrogenase (LDH) (> 270 U/L). Alanine transaminase and aspartate transaminase were elevated in 32 of 111 (28.8%) and 69 of 115 (60.0%) patients, respectively.

### Treatment and course of illness

Of all patients, 125 (80.6%) were treated with oseltamivir, although only 16 patients (10.3%) received antiviral therapy within 48 hours after symptom onset (Table 2). Broad-spectrum antibiotics were prescribed in 139 (89.7%) patients as empiric or definitive therapy.

**Table 2:** Hospitalized adult patients with 2009 Influenza A (H1N1) in Beijing: complications, definitive treatment, and supportive treatment

	Survivor (n = 128)	Nonsurvivor (n = 27)	Total (n = 155)	P value
Complications				
Acute respiratory failure	39 (30.5%)	23 (85.2%)	62 (40.0%)	0.003
Septic shock	1 (0.8%)	17 (63.0%)	18 (11.6%)	< 0.001
Acute renal failure	4 (3.1%)	11 (40.7%)	15 (9.7%)	< 0.001
Altered mental status	6 (4.7%)	14 (51.9%)	20 (12.9%)	< 0.001
Bacterial pneumonia	15 (11.7%)	13 (48.1%)	28 (18.1%)	< 0.001
Bloodstream infection	0	3 (11.1%)	3 (1.9%)	0.006
Other infections	0	5 (18.5%)	5 (3.2%)	< 0.001
Antiviral agents	107 (83.6%)	25 (92.6%)	132 (85.2%)	0.371
Oseltamivir treatment	101 (78.9%)	24 (88.9%)	125 (80.6%)	0.259
within 48 hrs of symptom onset	12 (9.4%)	4 (14.8%)	16 (10.3%)	0.507
Broad-spectrum antibiotics	113 (88.3%)	26 (96.3%)	139 (89.7%)	0.309
Corticosteroids	35 (27.4%)	17 (63.0%)	52 (33.5%)	< 0.001
Vasopressors	5 (3.9%)	23 (85.2%)	28 (18.1%)	< 0.001
Inotropes	2 (1.6%)	7 (25.9%)	9 (5.8%)	< 0.001
Neuromuscular blockade	1 (0.8%)	7 (25.9%)	8 (5.2%)	< 0.001
Noninvasive ventilation	19 (14.9%)	13 (48.1%)	32 (20.6%)	< 0.001
Invasive ventilation	19 (14.9%)	24 (88.9%)	43 (27.7%)	< 0.001
Continuous renal replacement therapy	4 (3.1%)	13 (48.1%)	17 (11.0%)	< 0.001

The most common organ failures during hospital stay included acute respiratory failure (40.0%), altered mental status (12.9%), septic shock (11.6%), and acute renal failure (9.7%). Forty-three patients (27.7%) were treated with invasive mechanical ventilation, while noninvasive mechanical ventilation was used in 32 patients (20.6%). Vasopressors and inotropes were used in 28 (18.1%) and 9 (5.8%) patients, respectively.

Fifty-two patients (33.5%) were treated with systemic corticosteroids. Daily dose of corticosteroids ranged from methylprednisolone 12 mg to 320 mg (or equivalent dose), with a median dose of 80 mg (interquartile range, 80 mg to 160 mg).

### Outcome and risk factors

**Figure 1:**
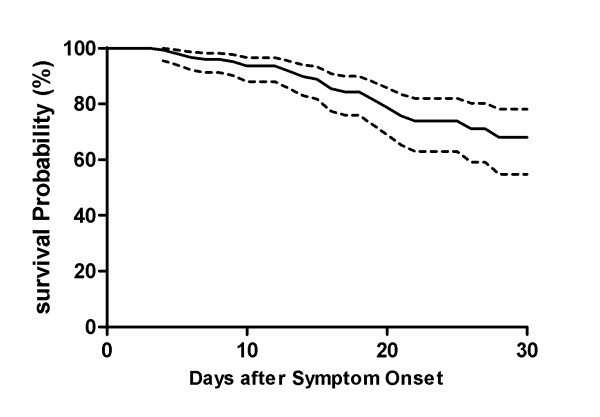
**Kaplan-Meier survival curve of hospitalized patients with 2009 influenza A (H1N1) infection**. (Dash lines represent 95% confidence interval).

Twenty-seven patients (17.4%) died during hospital stay, all within 28 days after admission (Figure 1). Causes of death included multiple organ failure (n = 14), refractory circulatory shock (n = 6), refractory respiratory failure (n = 5), and others (n = 2).

Compared with survivors, more nonsurvivors had hypertension (37.0% vs. 19.5%, p = 0.048). On hospital admission, nonsurvivors were more likely to be dyspneic (77.8% vs. 47.7%, p = 0.004), and lactate dehydrogenase (LDH) level was significantly higher (744 ± 759 vs. 356 ± 240 U/L, p = 0.041) (Table 1). During hospitalization, more nonsurvivors developed organ failure (acute respiratory failure, altered mental status, septic shock, and acute renal failure) and secondary infections (Table 2). Therefore, nonsurvivors were more likely to be treated with vasoactive agents, mechanical ventilation, and continuous renal replacement therapy (Table 2).

Risk factors for hospital mortality by multivariate logistic regression analysis included diabetes (odds ratio [OR] 8.830, 95% confidence interval [CI] 2.041 to 38.201, p = 0.004), and lactate dehydrogenase level (per 100 U/L increase) (OR 1.240, 95% CI 1.025 to 1.500, p = 0.027) (Table 3).

**Table 3:** Hospitalized adult patients with 2009 Influenza A (H1N1) in Beijing: risk factor for hospital mortality by logistic regression analysis

	OR	95%CI	P
Model 1			

Diabetes	8.830	2.041 - 38.201	0.004
LDH on admission, per 100 U/L increase	1.240	1.025 - 1.500	0.027
Corticosteroids treatment	3.668	0.987 - 13.640	0.052
			
Model 2			
Septic shock	143.039	10.355 - 1975.8	< 0.001
Altered mental status	8.482	1.760 - 40.887	0.008
Acute respiratory failure	9.982	0.947 - 105.240	0.056

CI, confidence interval; LDH, lactate dehydrogenase; OR, odds ratio

Variables included in model 1: ethnicity, comorbidity (hypertension, diabetes), symptoms at disease onset (dyspnea, sore throat), clinical presentation on admission (dyspnea), laboratory test on admission (lactate dehydrogenase), and corticosteroids treatment.

Variables included in model 2: complications (acute respiratory failure, septic shock, acute renal failure, altered mental status, bacterial pneumonia, bloodstream infection, other infections)

Oseltamivir treatment did not result in significant improvement in patient outcome (Table 2). Among 125 patients treated with oseltamivir, initiation of antiviral therapy within 48 hours of symptom onset showed no influence on hospital mortality (4/16 vs. 20/109, p = 0.508).

### Corticosteroids treatment

Corticosteroids treatment was associated with a trend toward higher hospital mortality (OR 3.668, 95% CI 0.987 to 13.640, p = 0.052) (Table 3). However, corticosteroids were used in more critically ill patients, as shown in Table 4. LDH level on hospital admission and acute respiratory failure were independent predictors of corticosteroids treatment (Table 4). Moreover, patients treated with a lower daily dose of corticosteroids (≤ 80 mg methylpredisolone or equivalent dose) exhibited a similar mortality rate compared with those treated with higher daily dose (9/30 vs. 8/22, p = 0.854). Subgroup analysis revealed that corticosteroids treatment was associated with higher hospital mortality rate in patients without acute renal failure (10/42 vs. 6/98, p = 0.0064) or altered mental status (8/41 vs. 5/94, p = 0.0242), and in patients with bacterial pneumonia (9/11 vs. 4/17, p = 0.0085). There was no systemic treatment effect on hospital mortality of either lower or higher daily dose of corticosteroids compared with no corticosteroids treatment (Additional file 1, Table S1).

**Table 4:** Comparison of patients treated with and without corticosteroids

	No corticosteroids (n = 103)	Corticosteroids (n = 52)	Univariate analysis OR (95%CI)	Multivariate analysis OR (95%CI)
Comorbidity				
Cardiovascular diseases	35	10	0.463 (0.208 - 1.031)	
Chronic lung diseases	20	2	0.116 (0.037 - 0.740)	
Symptoms at disease onset				
Dyspnea	40	33	2.736 (1.373 - 5.452)	
Hemoptysis	9	10	2.487 (0.942 - 6.568)	
On hospital admission				
Dyspnea	42	40	4.841 (2.275 - 10.304)	
Pneumonia on CXR	81	48	3.259 (1.060 - 10.025)	
Laboratory tests on admission				
CPK	260 ± 447	577 ± 795	1.001 (1.000 - 1.002)	
CK-MB	20 ± 30	32 ± 33	1.014 (0.997 - 1.032)	
LDH, per 100 U/L increase	305 ± 229	658 ± 572	1.508 (1.219 - 1.866)	1.401 (1.107 - 1.773)
Complications				
Acute respiratory failure	24 (23.3%)	38 (73.1%)	8.935 (4.160 - 19.187)	8.738 (2.504 - 30.489)
Septic shock	5 (4.9%)	13 (25.0%)	6.533 (2.183 - 19.551)	
Acute renal failure	5 (4.9%)	10 (19.2%)	4.667 (1.503 - 14.486)	
Altered mental status	9 (8.7%)	11 (21.2%)	2.842 (1.093 - 7.390)	
Bloodstream infection	0	3	1.061 (0.992 - 1.135)	
Other infections	1	4	8.500 (0.925 - 78.106)	
Supportive treatment				
Vasopresssors	9	19	6.013 (2.478 - 14.596)	
Neuromuscular blockade	1	7	15.867 (1.896 - 132.776)	
Noninvasive ventilation	11	21	5.666 (2.467 - 13.063)	
Invasive ventilation	16	27	5.873 (2.742 - 12.578)	

CK-MB, creatine kinase-MB; CPK, creatine phosphokinase; CXR, chest-X ray; LDH, lactate dehydrogenase

No interaction between corticosteroids treatment and any complications (including acute respiratory failure, septic shock, acute renal failure, altered mental status, bacterial pneumonia, bloodstream infection, and other infections) or oseltamivir treatment was found.

## Discussion

In this retrospective study of hospitalized adult patients with confirmed infection of 2009 influenza A(H1N1), we have found that diabetes, serum level of LDH on hospital admission, septic shock, and altered mental status were independent predictors of hospital mortality. In addition, corticosteroids treatment is associated with a trend towards worse outcome, although it is more likely to be used in critical illness, as shown by an elevated LDH level and the presence of acute respiratory failure.

The mortality seen in our study is significantly higher than those of hospitalized patients in the United States [7], United Kingdom [14], and Australia [15]. The later studies often summarized the initial hospitalized patients in each country, when even mild cases were admitted due to uncertainty of disease progression and prognosis. In contrary, the patients in our study developed the illness about 5 months after the first case of 2009 influenza A(H1N1) in China [8], when only high-risk patients were hospitalized, while most mild cases were followed up in fever clinics. The above difference in admission policy may be reflected by the significantly longer median time from symptom onset to hospital admission (5 vs. 2 to 3 days), the higher proportion of patients with signs of pneumonia on Chest X-ray (83.2% vs. 29 to 40%), and the higher mortality rate in our study, which is similar to those observed in patients requiring intensive care support in Australia [16] and Canada [10].

Almost half of the 155 hospitalized patients in our analysis do not have any comorbidities. The absence of serious comorbidities emphasizes that young, relatively healthy adults were the primary population affected by 2009 influenza A(H1N1) infection. On the contrary, underlying medical conditions associated with complications from seasonal influenza have been consistently shown as risk factors for hospital admission [17], intensive care unit (ICU) admission [18,19], and death [17,19,20]. The commonly acknowledged comorbidities may be classified according to chronic medical conditions recognized by the Advisory Committee on Immunization Practices, including cardiovascular disease, pulmonary disease, liver disease, cancer, and diabetes [17]. Diabetes and stress-induced hyperglycemia are known to be associated with an increased risk of complications and death among critically ill patients [21]. In this study, when infected with 2009 influenza A(H1N1), patients with diabetes were at a higher risk of death, compared with those without diabetes. In a cohort of 1479 patients admitted to hospital with laboratory-confirmed pandemic (H1N1) influenza in Canada, Campbell reported that the risk of a severe outcome (including death and ICU admission) was greatest among patients with diabetes (relative risk 2.2, 95%CI 1.7 - 2.7) [18]. Likewise, when comparing 1266 hospitalized patients with the general population in France, Hanslik also found that diabetes was significantly associated with death (OR 3.5, 95%CI 2.5 - 5.1) [19].

Patients with increased serum level of LDH on hospital admission are at a higher risk of death during hospitalization. This is not a surprising finding, because, as a non-specific enzyme found ubiquitously in cells, the increased serum level of LDH probably indicates the degree of tissue necrosis, and hence the severity of the viral infection as well as pneumonia. The association of high level of LDH and severity of illness, or even death, although not found in patients with 2009 influenza A(H1N1), has been widely reported in patients with severe acute respiratory syndrome (SARS). For example, high LDH level on hospital admission was associated with development of acute respiratory distress syndrome [22], and death during hospital stay [23,24].

It is recommended that therapy with a neuraminidase inhibitor is especially important for patients with underlying risk factors and those with severe or progressive clinical illness [25]. However, we did not observe any treatment effect of oseltamivir in our analysis. Only 16 out of 155 patients (10.3%) in our study received oseltamivir treatment within 48 hours after symptom onset. In comparison, Jain and Louie found that early antiviral therapy was administered in 39% and 51% of patients, respectively [7,26]. Studies have shown that delayed antiviral treatment is associated with high risk of progression to severe disease such as ICU admission and respiratory failure, prolonged hospital stay, and even death [25,27]. It is also possible that the delay in antiviral treatment in our patients resulted in higher hospital mortality (17.4%) compared with the subgroup of adult hospitalized patients with 2009 influenza A(H1N1) in the United States [7,26].

Corticosteroids remain a controversy in the treatment of viral infection [28]. WHO strongly recommends that systemic corticosteroids should not be given, unless indicated for other reasons or as part of an approved research protocol [29]. In a retrospective study of 78 consecutive adult SARS patients, corticosteroids treatment was associated with a 20.7-fold increase in either ICU admission or mortality, independent of age and disease severity [30]. Liem et al also found that corticosteroids treatment was an independent risk factor of death in human H5N1 influenza A infection [31]. Observational studies demonstrated that corticosteroid use was associated with slower viral clearance, significantly increased odds of persistent viral replication 7 days after symptom onset [32], and a longer duration of viral shedding with increased corticosteroid dose [33]. In patients with 2009 influenza A(H1N1) infection, Jain et al found that steroids were more commonly used in patients with severe outcome compared with those without severe outcome (52% vs. 31%, p < 0.05) [7]. Our data suggested that clinicians tend to use corticosteroids in more critically ill patients, such as patients with elevated LDH level or acute respiratory failure. However, even after adjusted for these confounding factors, results of logistic regression analysis still indicated a trend towards fatal outcome associated with corticosteroid use.

Our study is subject to several limitations. Case capture was based on passive reporting by clinicians, and underreporting may have occurred because of poor recognition and the lack of specificity of clinical presentations. The retrospective nature of this study precluded the possibility of integrating body mass index into our regression analysis model, as height and weight data were not routinely documented in most cases. However, the effect of underlying medical conditions and morbid obesity may not be independent. For example, diabetes is reportedly up to seven times more prevalent in obese persons [34], so that the diabetic cases were most likely among obese patients. The relatively large odds ratio for diabetes for hospital mortality in our study suggests an additional risk compared to obesity alone.

## Conclusions

We have found that hospitalized patients with 2009 influenza A(H1N1) have relatively poor outcome. Diabetes and elevated LDH level on hospital admission, as well as the presence of septic shock and altered mental status, represent independent risk factors for hospital death among these patients. Early recognition of these risk factors may help clinicians to identify the high-risk patients. In addition, corticosteroids use should not be regarded as routine pharmacologic therapy, although this may need confirmation by further large scale prospective study.

## Competing interests

No external funding was received in support of this study. None of the authors have professional, personal, or financial conflicts of interest to report.

## Authors' contributions

BD conceived of the study, participated in its design and coordination, had full access to all of the data in the study and takes responsibility for the integrity of the data and the accuracy of the data analysis. XX conceived of the study, and participated in its design and coordination, and helped to draft the manuscript. YX participated in the design of the study, and helped to draft the manuscript. LJ participated in the design of the study, and helped to draft the manuscript. AL participated in the design of the study, and helped to draft the manuscript. JD participated in the design and coordination of the study, and helped to draft the manuscript. All authors reviewed and approved the final manuscript.

## Pre-publication history

The pre-publication history for this paper can be accessed here:

http://www.biomedcentral.com/1471-2334/10/256/prepub

## Supplementary Material

Additional file 1 **Supplementary table S1**. Table to show subgroup analysis of the effect of corticosteroids treatment on hospital mortality. Click here for file
