# Supplementary material for: Hospitalized adult patients with 2009 influenza A(H1N1) in Beijing, China: risk factors for hospital mortality
Source: BMC Infect Dis. 2010 Aug 27;10:256. doi: 10.1186/1471-2334-10-256 (PMC2941683; doi:10.1186/1471-2334-10-256)
Supplement: Additional file 1 — Supplementary table S1. Table to show subgroup analysis of the effect of corticosteroids treatment on hospital mortality. [file 1471-2334-10-256-S1.DOC]

# Hospitalized adult patients with 2009 influenza A(H1N1) in Beijing, China: risk factors for hospital mortality

**Additional File 1**

Title: Supplementary table S1

Description: Table to show subgroup analysis of the effect of corticosteroids treatment on hospital mortality

Table S1. Effect of corticosteroids treatment on hospital mortality: result of subgroup analysis

|  | No corticosteroids  (n = 103) | Corticosteroids¶ | | |
| --- | --- | --- | --- | --- |
|  |  | Low-dose  (n = 30) | High-dose  (n = 22) | Total  (n = 52) |
| Acute respiratory failure |  |  |  |  |
| Yes (n = 62) | 33%  (8/24) | 42%  (8/19) | 37%  (7/19) | 39%  (15/38) |
| No (n = 93) | 3%  (2/79) | 9%  (1/11) | 33%  (1/3) | 14%  (2/14) |
| Septic shock |  |  |  |  |
| Yes (n = 18) | 100%  (5/5) | 85%  (6/7) | 100%  (6/6) | 92%  (12/13) |
| No (n = 137) | 5%  (5/98) | 13%  (3/23) | 13%  (2/16) | 13%  (5/39) |
| Acute renal failure |  |  |  |  |
| Yes (n = 15) | 80%  (4/5) | 50%  (1/2) | 75%  (6/8) | 70%  (7/10) |
| No (n = 140) | 6%  (6/98) | 29%**  (8/28) | 14%  (2/14) | 24%**  (10/42) |
| Altered mental status |  |  |  |  |
| Yes (n = 20) | 56%  (5/9) | 50%  (2/4) | 100%  (7/7) | 82%  (9/11) |
| No (n = 135) | 5%  (5/94) | 27%**  (7/26) | 7%  (1/15) | 20%*  (8/41) |
| Bacterial pneumonia |  |  |  |  |
| Yes (n = 28) | 24%  (4/17) | 67%  (2/3) | 87%**  (7/8) | 91%**  (9/11) |
| No (n = 127) | 7%  (6/86) | 26%*  (7/27) | 7%  (1/14) | 20%  (8/41) |

*p < 0.05, **p < 0.01 vs. no corticosteroids

¶low-dose refers to daily dose of corticosteroids ≤ 80 mg methylpredisolone or equivalent dose, while high-dose refers to daily dose of corticosteroids > 80 mg methylpredisolone or equivalent dose.
